# Highly‐Polarized Near‐Infrared Photodetector Based on 2D Organic/Inorganic Van Der Waals Heterostructure

**DOI:** 10.1002/advs.202508332

**Published:** 2025-08-13

**Authors:** Wen Xu, Shuchao Qin, Qianqian Du, Yongji Zhang, Yanxun Zhang, Wenjun Wang, Fengqiu Wang

**Affiliations:** ^1^ School of Physical Science and Information Engineering Liaocheng University Liaocheng 252059 China; ^2^ National Laboratory of Solid State Microstructures and Jiangsu Provincial Key Laboratory of Advanced Photonic and Electronic Materials School of Electronic Science and Engineering Nanjing University Nanjing 210093 China

**Keywords:** graphene, heterostructure, near‐infrared photodetector, TiOPc single crystal

## Abstract

Organic semiconductors have demonstrated exceptional performance due to their inherent advantages such as simple processability, and superior mechanical properties. Developing polarization‐sensitive near‐infrared (NIR) organic photodetectors is crucial for their application in target recognition, biological imaging, and wearable optoelectronics. However, high‐performance NIR photon detection still faces challenges for organic materials, due to their intrinsic limitations including low carrier mobility, and poor exciton dissociation. Here, a polarization‐sensitive NIR photodetector is demonstrated with a linear dichroic ratio of 5.3, employing a two‐dimensional (2D) TiOPc single‐crystal/graphene heterostructure. Remarkable absorption, optimized exciton diffusion of single crystal, and efficient interfacial charge transfer enable a high NIR responsivity of > 10^3^ A W^−1^ and specific detectivity of 10^10^ Jones for 980 nm irradiation, with a reasonable −3 dB bandwidth of >1 kHz. Under 850 nm illumination, it exhibits an even higher responsivity of > 10^4^ A W^−1^ and specific detectivity of > 10^11^ Jones, attributed to stronger absorption. This NIR responsivity represents a tenfold improvement over existing organic polarization photodetectors. Finally, the high‐resolution polarization‐dependent single‐pixel imaging in NIR range is achieved, highlighting its great potential for image recognition applications. This work opens new avenues for exploiting high‐performance NIR polarized photodetectors.

## Graphical abstract

Employing 2D organic/inorganic van der Waals heterostructure, a highly sensitive polarized near‐infrared photodetector is fabricated. This device demonstrates exceptional visible and NIR detection capabilities, achieving a responsivity as high as 10^4^ A W^−1^. By leveraging the pronounced anisotropy of TiOPc single crystals, the device exhibits excellent NIR polarization detection performance.

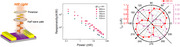

## 1 Introduction

Photodetectors, serving as cornerstone components of modern photoelectric systems, play an indispensable role in diverse fields such as optical communication, environmental sensing, and high‐resolution imaging.^[^ ^1^, ^2^ ^]^ In recent years, polarization‐resolved detection technology has garnered significant attention due to its capability to analyze the Stokes parameters of light across a wavelength range.^[^ ^3^, ^4^ ^]^ By acquiring the polarization state information of target objects, polarization‐sensing can significantly enhance imaging contrast and target recognition accuracy in complex environments, thereby demonstrating unique advantages in areas like biomedical diagnosis and military reconnaissance. Particularly in the NIR band (750‐2500 nm), the distinctive tissue penetrability and eye safety features of this spectral region enable polarization‐sensitive NIR detection technology to provide innovative solutions for non‐invasive biological imaging and all‐weather environmental monitoring.^[^ ^5^, ^6^, ^7^ ^]^ The polarization detector could enable novel applications in multidimensional information transmission and intelligent vision systems, such as polarization‐encoded optical communication. And the polarization state modulation technology could introduce a new layer of security for optical information encryption. In addition, analyzing the polarization characteristics of rock types in geological remote sensing can improve exploration accuracy.^[^ ^8^, ^9^, ^10^, ^11^ ^]^ Therefore, to achieve high photoresponsivity and sensitivity, the narrow bandgap materials (<1.6 eV) are essential for exciton generation.

Traditional near‐infrared polarization detectors primarily utilize established inorganic semiconductor‐based systems including bulk silicon, InGaAs, and InP material systems, which are constrained by their intrinsic narrow bandgap characteristics.^[^ ^12^, ^13^ ^]^ However, these detectors face several limitations, including high fabrication expenditures, limited structural adaptability, and thermal instability, which are incompatible with future low‐cost and wearable optoelectronic devices.^[^ ^14^ ^]^ In addition, these devices typically lack polarization‐sensitive detection capabilities due to their optically isotropic nature in standard configurations.^[^ ^15^ ^]^ Organic semiconductors represent viable alternatives due to their easy processability, flexibility, and tunable bandgap engineering capabilities, offering new opportunities to develop the advanced photodetectors.^[^ ^16^, ^17^ ^]^ However, high‐performance NIR organic photodetectors remain scarce to the best of our knowledge, as most organic semiconductors exhibit excessively wide bandgaps. Phthalocyanine complexes (MPc), characterized by their highly conjugated macrocyclic structures and adjustable electronic properties, have attracted significant attention by virtue of their superior absorption and carrier mobility. Notably, non‐planar phthalocyanine derivatives, such as titanium oxophthalocyanine (TiOPc), exhibit exceptional NIR absorption properties.^[^ ^18^, ^19^, ^20^ ^]^

Despite these advantages, organic matrix suffers from the high binding energy of Frenkel excitons that is always much higher than that of thermal energy, which confines the exciton dissociation.^[^ ^21^, ^22^, ^23^ ^]^ Blended donor/acceptor interface in hybrid organic film, forming a built‐in electric field, is an effective solution to overcome the binding energy.^[^ ^16^, ^24^ ^]^ Unfortunately, such strategies often yield polarization‐insensitive devices, as most hybrid films exist in an amorphous state. Furthermore, the carrier mobility of amorphous organic film is inferior, restricting the quantum efficiency of organic devices. In contrast, organic single crystal structure constructed by π‐conjugated molecules exhibit long‐range periodic order, enabling anisotropy, better exciton diffusion, and efficient charge transport.^[^ ^25^, ^26^ ^]^ Previous studies have reported polarized photodetectors based on single‐crystal structures,^[^ ^27^, ^28^, ^29^, ^30^ ^]^ while the most response band focus on the visible and short‐wave infrared range. Li at al. reported an NIR polarized photodetector based on CuPc single crystal, showing a responsivity of 200 A W^−1^ at 785 nm but with a low linear dichroic ratio.^[^ ^31^ ^]^ By virtue of the high quality of single crystal, a sensitive DPA‐based photodetector was demonstrated in Wang's work, and the device shows a high responsivity of 10^4^ AW^−1^, while the linear dichroic ratio is lower than 2. Recently, the dichroic ratio is raised up to 4.26 in BPTTE single crystal by Dong's research group, while the spectral response is limited to the ultraviolet (UV) band.^[^ ^32^ ^]^

Here, we successfully prepared highly stable TiOPc single crystals with pronounced in‐plane anisotropy using micro‐spacing air sublimation technology. These crystals exhibit broadband infrared absorption in the spectral range of 300–1600 nm. By constructing a TiOPc/graphene heterostructure, we designed and fabricated a high‐performance polarization‐sensitive NIR photodetector, achieving a remarkable linear dichroic ratio of 5.3, which is one of the highest values among current organic polarization‐sensitive photodetectors. Leveraging the enormous absorption and excellent exciton diffusion of TiOPc single crystal, the high carrier mobility of graphene, and efficient exciton separation and charge transfer at TiOPc/graphene interface, a good infrared responsivity of > 10^3^ A W^−1^ @ 980 nm (> 10^4^ A W^−1^ @ 850 nm) and specific detectivity of 10^10^ Jones @ 980 nm (10^11^ Jones @ 850 nm) were realized. Compared with existing inorganic photodetectors, our device exhibits > 10^2^× higher responsivity across visible and NIR wavelength range.^[^ ^33^, ^34^, ^35^ ^]^ Even for the 1550 nm laser illumination, the responsivity remains at 32 A W^−1^. This device exhibits a reasonable −3 dB bandwidth > 1 kHz for NIR illumination. This performance characteristic can be attributed to the minimal charge trap concentration of single crystal and its well‐optimized heterojunction structure. Additionally, several high‐definition polarization‐selective single‐pixel imaging were successfully demonstrated in NIR range. These results not only highlight the potential of high‐quality organic single crystals for next‐generation polarized photodetectors, but also establish key design principles for advancing near‐infrared photodetection systems.

## 2 Results and Discussion

**Figure 1:**
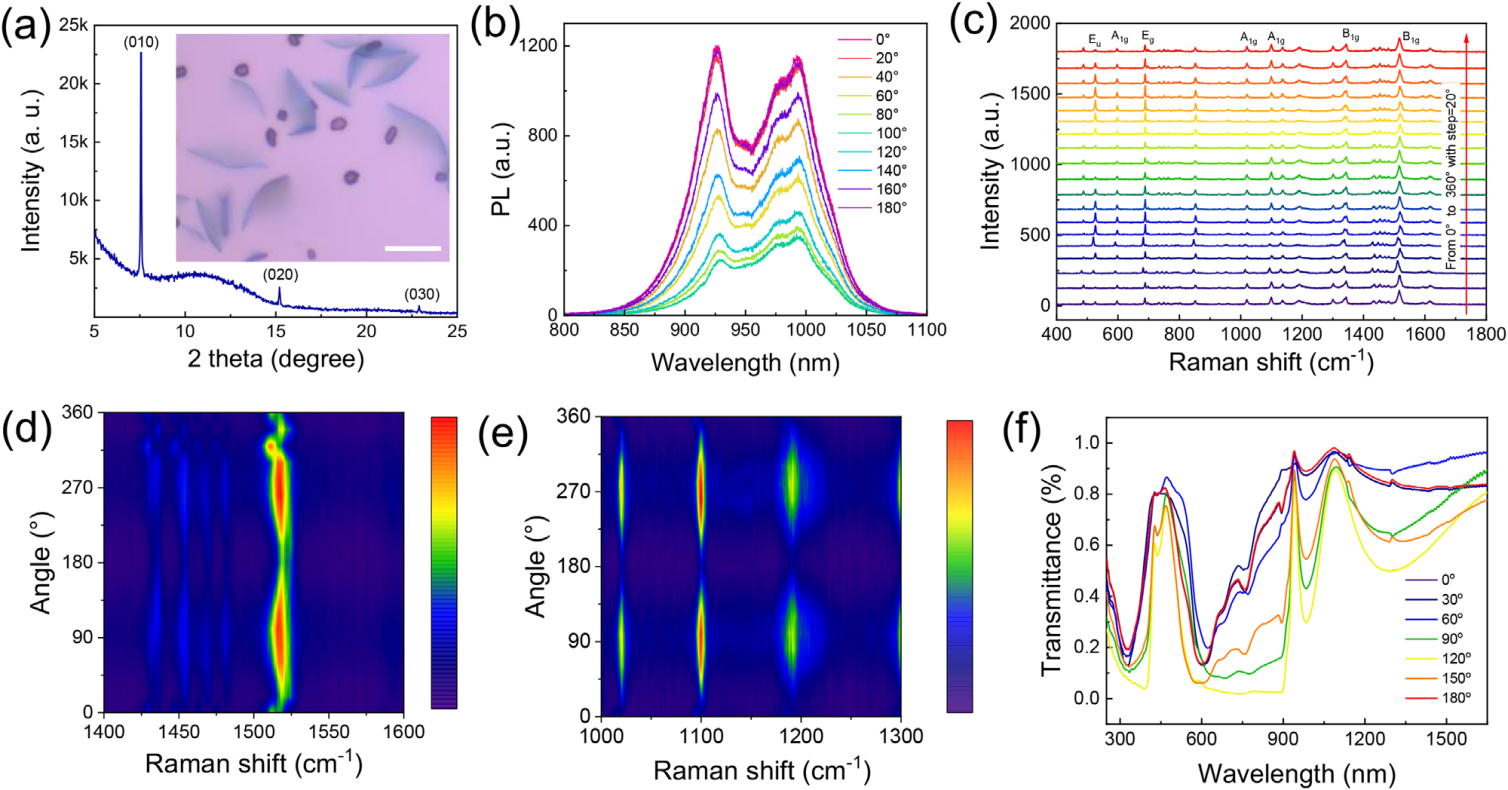
Characterization of TiOPc single crystal and its anisotropy. a) XRD pattern of a TiOPc single crystal. Inset presents the optical microscope image of a TiOPc single crystal. Scale bar: 20 µm. b) PL spectra of TiOPc single crystal under different polarization angles. c) Raman spectra of TiOPc single crystal under different polarization angles. d) The contour color map of Raman spectra of TiOPC crystal in the range of 1400–1600 cm^−1^. e) The contour color map of Raman spectra in the range of 1000–1300 cm^−1^. f) The transmission spectra of a TiOPc crystal under different polarization angles.

We successfully synthesized high‐quality single crystals of TiOPc using the micro‐spacing air sublimation method, and the typical optical image is shown in the inset of **Figure** **1a**. The majority of the samples exhibit upright growth, which is advantageous for the subsequent sample transfer processing. To further validate the molecular packing within the single crystal, X‐ray diffraction (XRD) analysis was also performed in Figure 1a. The XRD result shows several prominent peaks at 7.58°, 15.22°, and 22.92°, corresponding to the diffraction from (010), (020), and (030) lattice planes, respectively.^[^ ^36^ ^]^ The polarized optical microscopy (POM) demonstrated the anisotropic nature of the TiOPc crystal, as shown in Figure S1 (Supporting Information). The POM images of TiOPc crystal sample illuminated by a 470 nm Light‐Emitting Diode (LED) light source reveal uniform brightness across the crystal interface. The angular alignment of the crystal's principal axis relative to the polarization direction of the incident light is a critical factor in determining the variation in sample brightness, thus offering compelling evidence for the intrinsic anisotropic monocrystalline nature of TiOPc. To further verify the anisotropic optical properties of TiOPc crystals, polarization‐resolved photoluminescence (PL) spectra were measured at various polarization angles (ranging from 0° to 180° with a step size of 20°), as shown in Figure  1b. A prominent peak at 926 nm and a shoulder peak at 995 nm are observed, indicating that the TiOPc crystal exhibits high purity with minimal deep trap states. With the variation of polarization angle, the PL intensity exhibits periodic variations, indicating the pronounced anisotropic optical behavior. Polar plots were constructed using the intensities of two main emission peak intensity, revealing polarization ratios of 5.3 at 926 and 3.5 at 995 nm, as presented in Figure S2 (Supporting Information). The Raman effect arises from molecular vibrations (or lattice vibrations), and experimental findings indicate that Raman intensity depends on the orientation (crystal plane index) of the single crystal. We investigated the Raman signal of a TiOPc single crystal on graphene using a 532 nm excitation laser. A characteristic peak was observed at 1517 cm^−1^, corresponding to the pyrrole ring stretching mode. This feature serves as a well‐established hallmark of TiOPc crystalline structures.^[^ ^36^, ^37^ ^]^ All other peaks exhibited high intensity, indicating excellent crystallinity and structural regularity of the crystal lattice. Polarization‐resolved Raman spectra were acquired in the range of 400–1800 cm^−1^ from 0 to 360° (Figure 1c). Based on previous studies, TiOPc demonstrates sensitivity to light polarization within the spectral regions of 1000–1150 and 1400–1650 cm^−1^.^[^ ^38^ ^]^ Consequently, we analyzed the polarization characteristics of these two bands in Figure 1c and generated the 2D contour color maps, as shown in Figure 1d,e. These results reveal significant intensity variations of certain Raman peaks as the polarization angle changes. This behavior also confirms the anisotropic optical properties of TiOPc crystal. In addition, the uniform Raman and PL mapping signals confirm the high homogeneity of the grown TiOPc single crystal (Figure S3a, Supporting Information). Figure 1f displays the visible‐near‐infrared transmission spectrum of a TiOPc single crystal transferred onto a quartz substrate. The strong absorption in the NIR region highlights its potential in NIR photon detection, supported by the prominent absorption peak in this spectral range. In the NIR region, the absorption of the TiOPc single crystal exhibits significant dependence on the incident direction of polarized light, which is particularly important for polarization detection.

**Figure 2:**
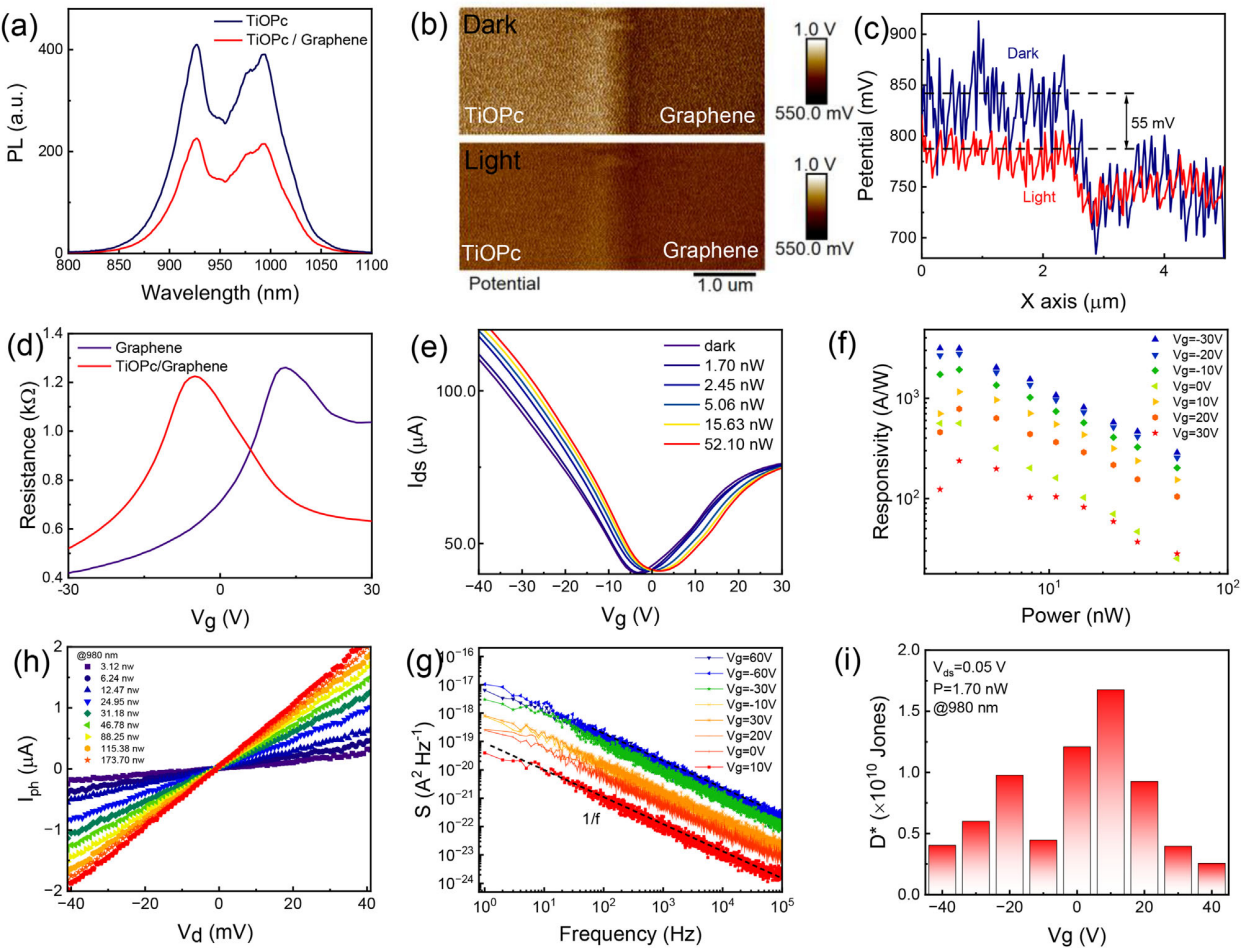
Optical gating in the TiOPc‐graphene interface. a) PL of TiOPc single crystal with (red) and without (blue) graphene. b) KPFM images of the interface between TiOPc and graphene under dark (upper) and 980 nm laser irradiation (bottom), respectively. c) The potential profile difference under light and dark conditions. d) Transfer curves of TiOPc/graphene transistor compared with that of pristine graphene transistor. e) Transfer curves of TiOPc/graphene transistor under different optical powers (λ = 980 nm, V_d_ = 50 mV). f) Responsivities as a function of light powers under different gate voltages (λ = 980 nm, V_d_ = 50 mV). g) Dependence of the photocurrent on the source‐drain voltage (λ = 980 nm). h) Noise current spectral density as a function of frequency under different gate bias. i) The specific detectivity (D*) as a function of gate voltage (V_g_) at finite illumination (λ = 980 nm, P = 1.70 nW, V_ds_ = 0.05 V).

Subsequently, the crystal was transferred onto a high‐quality graphene field‐effect transistor (FET) device via PDMS‐assisted transfer technology, fabricating an asymmetric organic heterostructure device, in which graphene serves as the carrier transport layer and the TiOPc crystal functions as the light absorption layer. The transfer process does not induce structural damage to the crystal, as proven by Raman and PL mappings in Figures S3b and S4 (Supporting Information). The optical microscope image of the fabricated device is provided in Figure S5a (Supporting Information). In Figure 2a, the photoluminescence (PL) spectra of the sample at various positions are presented. By comparing the two PL spectra, it is evident that the presence of graphene beneath the TiOPc layer leads to PL quenching (reduced by ≈50%), which suggests that a significant number of exciton dissociations occur at the TiOPc/graphene interface.^[^ ^39^ ^]^ This phenomenon is crucial for enhancing the optoelectrical performance and sensitivity of the device. The interfacial charge transport dynamics and energy band alignment characteristics of the TiOPc/graphene heterostructure were quantitatively investigated using Kelvin probe force microscopy (KPFM, Figure 2b), with complementary support from in situ atomic force microscopy (AFM) surface morphological analysis (Figure S6a,b, Supporting Information). The contact potential difference (V_CPD_) reveals a potential offset of 102 mV at the TiOPc/graphene interface (Figure S6c,d, Supporting Information), suggesting that the Fermi level of graphene is lower than that of TiOPc. This electronic configuration results in the upward bending of the interface energy band within the heterostructure (Figure S5b, Supporting Information), inducing a built‐in electric field and facilitating the directional migration of photogenerated holes from TiOPc to the graphene layer. Owing to the gate‐tunable work function properties of graphene, the intensity of the internal electric field can be modulated by gate bias and light irradiation. We initially explored the modulation of the built‐in field intensity through light irradiation. Under light exposure, the built‐in field facilitates the dissociation of excitons. Consequently, the built‐in field under illumination also be weakened. To confirm this supposition, KPFM measurements were conducted under 980 nm NIR laser irradiation, as depicted at the bottom of Figure 2b. Under illumination, the built‐in electric field at the TiOPc/graphene interface was investigated and compared with that under dark conditions (see Figure 2c). A V_CPD_ signal difference of 55 mV was detected, highlighting the crucial role of the built‐in electric field in achieving high sensitivity. The comparative analysis of the transfer curves between pristine graphene and graphene/TiOPc heterostructure reveals that the Dirac point of the heterostructure device shifts to the left by 18 V (Figure 2d). This shift confirms the electron transfer from the TiOPc crystal to the graphene. Based on Equation n = Δ*V* × *C_g_*/*e*, where C_g_ denotes the capacitance per unit area and e represents the elementary charge, we estimated the electron doping concentration to be ≈ 10^12^ cm^−2^. This electron doping is in accordance with the upward bending of the energy levels near the TiOPc/graphene heterojunction. As shown in Figure 2e, this upward‐bending energy level facilitates the dissociation of excitons under illumination, driving photogenerated holes from TiOPc crystals into the graphene layer while retaining the electrons within the TiOPc crystals. Consequently, the Dirac point shifts to the right, forming a photocurrent. With increasing optical power under 980 nm laser excitation, the Dirac point exhibits a further rightward shift in Figure 2e. In order to evaluate the photoresponsivity to incident optical signals, specifically its ability to convert optical signals into electrical signals, we calculated the responsivity of the device at 980 nm using the formula $R=\frac{I_{ph}}{P_{in}}=\frac{|I_{light}-I_{dark}|}{P_{in}}$, as presented in Figure 2f. At V_ds_ = 50 mV, the NIR photoresponsivity of the device reaches over 3 × 10^3^ A W^−1^ for 980 nm irradiation, which significantly surpasses the performances of previously reported near‐infrared TiOPc‐based photodetectors.^[^ ^40^, ^41^, ^42^ ^]^ Similar to most graphene‐related photodetectors, the responsivity decreases with increasing optical power. This phenomenon can be attributed to three factors: first, the optical saturation absorption of the TiOPc single crystal; second, the reduction of the built‐in field pointing toward graphene near the heterojunction (as verified in Figure 2b); and thirdly, the increased recombination probability of non‐equilibrium carriers under illumination, which shortens the lifetime of photoexcited carriers. In Figure 2g, the functional relationship between the photocurrent (I_ph_) and the bias voltage (V_ds_) under various optical powers is illustrated. It is clear that there is a strong linear relationship between the I_ph_ and V_ds_, indicating that the higher responsivity can be achieved by applying a larger bias. Furthermore, the specific detectivity (D*, measured in units of cm Hz^1/2^ W^−1^) serves as a critical parameter for assessing the sensitivity of photodetectors. It quantifies the ratio of the signal detected under specified conditions to the corresponding noise level. To evaluate the signal‐to‐noise ratio (SNR) and specific detectivity of the device in terms of sensitivity, we conducted spectral noise density measurements across a range of gate voltages from ‐60 to 60 V. These measurements were performed to analyze the noise equivalent power (NEP, expressed in units of cm Hz^−1/2^): $NEP=\frac{\sqrt{\bar{{I_{n}}^{2}}}}{\sqrt{\Delta f}R}$.^[^ ^43^ ^]^ As depicted in Figure 2h, all low‐noise spectra exhibit a characteristic 1/*f* power density distribution spanning both low and high frequencies over the entire operational frequency range. This intrinsic noise behavior is primarily attributed to stochastic fluctuations in either charge carrier density or transport mobility parameters. Confined to a spectral resolution of Δ*f* = 1 Hz, the specific detectivity D* follows the analytical expression: $D^{*}=\frac{\sqrt{A}}{NEP}\approx \frac{R\sqrt{A}}{\sqrt{S(f)}}$, where A represents the active area of the device. Leveraging these noise density diagrams, it was accurately determined that at V_g_ = 10 V, the D* value at the 980 nm wavelength band reaches 1.6 × 10^10^ Jones, as illustrated in Figure 2i. We conducted a comprehensive series of measurements and calculations on the shorter 850 nm laser wavelength, revealing that the responsivity of the 850 nm band reached 1.9 × 10^4^ A W^−1^, with D* achieving 8.2×10^11^ Jones, as depicted in Figure S7 (Supporting Information). To evaluate the visible‐near‐infrared broadband performance of the device, additional measurements and calculations were performed at wavelengths of 532, 658, and 785 nm (as shown in Figure S8, Supporting Information). It was observed that under excitation by a 785 nm laser, the device exhibited a responsivity exceeding 10^3^ A W^−1^. Notably, both of responsivities exceeded 10^4^ A W^−1^ for the 532 and 658 nm lasers. Furthermore, the responsivity measurements of the other devices in the near‐infrared range (785, 850, and 980 nm) were also conducted, confirming values exceeding 10^3^ A W^−1^, as shown in Figure S9 (Supporting Information). This heterostructure device exhibits significantly enhanced responsivity and detectivity across the visible to NIR spectrum, with values 1 to 3 orders of magnitude higher than those of other organic polarization detectors.^[^ ^31^, ^44^, ^45^ ^]^ These results collectively demonstrate that this strategy of TiOPc/graphene heterostructure is superior for near‐infrared photon detection.

**Figure 3:**
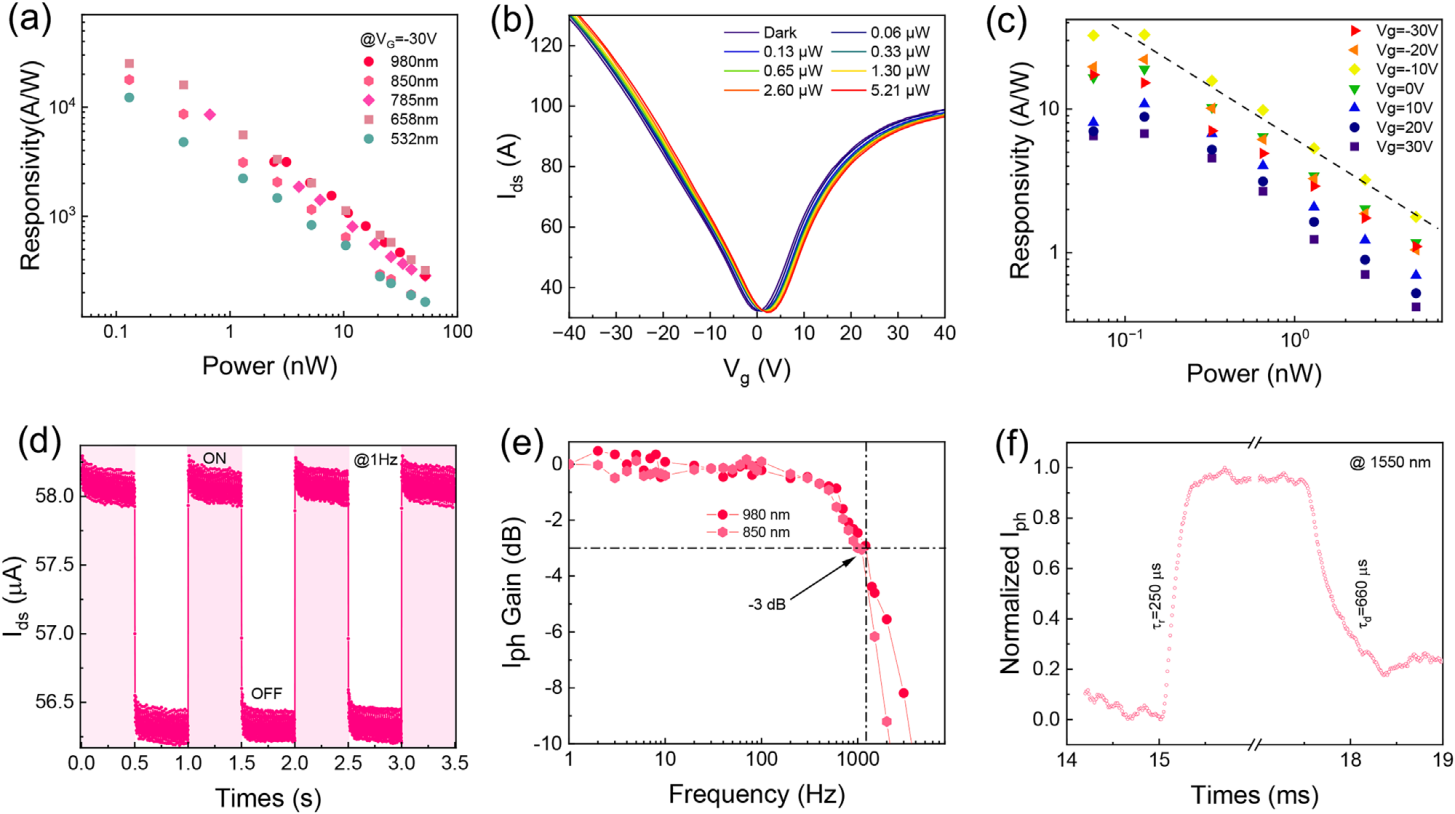
The photoelectric properties of TiOPc/graphene heterojunction. a) Responsivities as a function of optical power for several visible and NIR laser wavelengths (532, 658, 785 and 980 nm). b) Transfer curves of TiOPc/graphene transistor under different optical powers (λ = 1550 nm, V_d_ = 50 mV). c) Responsivities as a function of light powers under different gate voltages (λ = 1550 nm, V_d_ = 50 mV). d) Temporal photoresponse at 1 Hz modulated frequency (λ = 980 nm, V_d_ = 50 mV). e) Photocurrent gain expressed in dB as a function of the increasing light‐modulate frequency. f) The rise and decay times of the normalized photocurrent (λ = 1550 nm).

To systematically assess the broad‐spectrum detection capabilities of this device, its spectral response characteristics were evaluated under a gate bias of V_g_ = ‐30 V. Visible light laser sources (532 and 658 nm) and NIR laser sources (785, 850, and 980 nm) were used for this characterization, as presented in **Figure** **3**a. All responsivity of the device can reach up to 10^3^ AW^−1^ for all laser irradiation. Notably, the device exhibits a decent response at the 1550 nm communication wavelength. Figure 3b illustrates the transfer curves under 1550 nm illumination, at V_ds_ = 50 mV. It is evident that the photocurrent of the device increases significantly with increasing optical power. Using the changes of transfer curves in Figure 3b, we calculated the responsivity as the function of light power at different gate voltages in Figure 3c. Notably, under an illumination intensity of 0.06 µW, the peak responsivity of this system reaches 32 A W^−1^ at V_g_ = ‐10 V, surpassing the benchmark values of current near‐infrared light detection standards operating at a wavelength of 1550 nm. To accurately assess the sensitivity of the device, we examined its time‐dependent optical response under a 1 Hz optical modulation signal at 980 nm. When the incident light irradiates the entire device, stable ON/OFF switching characteristics are clearly observed in Figure 3d, indicating excellent optical switching ability and reversibility. To evaluate the stability of the device, we carried out 15 h continuous switching operation under 980 nm irradiation, in ambient condition (≈50% humidity). As shown in the Figure S10 (Supporting Information), its performance remains nearly unchanged under continuous 2 h illumination operation. Even after 15 h continuous irradiation, the photoresponse exhibited only a 15% degradation, indicating that the short‐term stability of the proposed device is satisfactory. Temporal response parameters (τ_r_ and τ_d_) are operationally defined using the photoresponse kinetics. Specifically, rise time τ_r_ represents the time interval required for the photocurrent to evolve from 10% to 90% of its saturation value. Conversely, decay time τ_d_ denotes the duration process of the photocurrent to decay from 90% to 10%. Temporal response analysis of the dynamic photoresponse in Figure S11 (Supporting Information) determined that rise/fall times (τ_r_/τ_d_) are about 380/540 µs and 410/360 µs for 850 and 980 nm laser illumination, respectively. We conducted a systematic spectral responsivity mapping to determine the frequency‐dependent photoconductive gain curves. In addition to characterizing the response speed on the time scale, a comprehensive full‐spectrum modulation transfer function analysis was also carried out, as illustrated in Figure 3e. The ‐3 dB bandwith is typically defined as the modulation frequency of the input optical signal at which the photocurrent decreases to 1/√2 of its original value. The cut‐off frequency at −3 dB is >1 kHz for both 850 and 980 nm illumination, indicating that the device can detect high‐frequency pulsed optical signals. Even for 1550 nm laser irradiation, the rise/decay times can be as low as 250/660 µs, as shown in Figure 3f. Compared to reported inorganic polarization detectors, our device exhibits a 100‐fold improvement in NIR responsivity, with comparable response speed. The detailed parameters comparison with high‐performance inorganic‐based photodetectors in recent literature are summarized in Figure S12 (Supporting Information), in which the overall performance of our device is very competitive.^[^ ^3^, ^4^, ^5^, ^6^, ^7^, ^46^, ^47^, ^48^ ^]^

**Figure 4:**
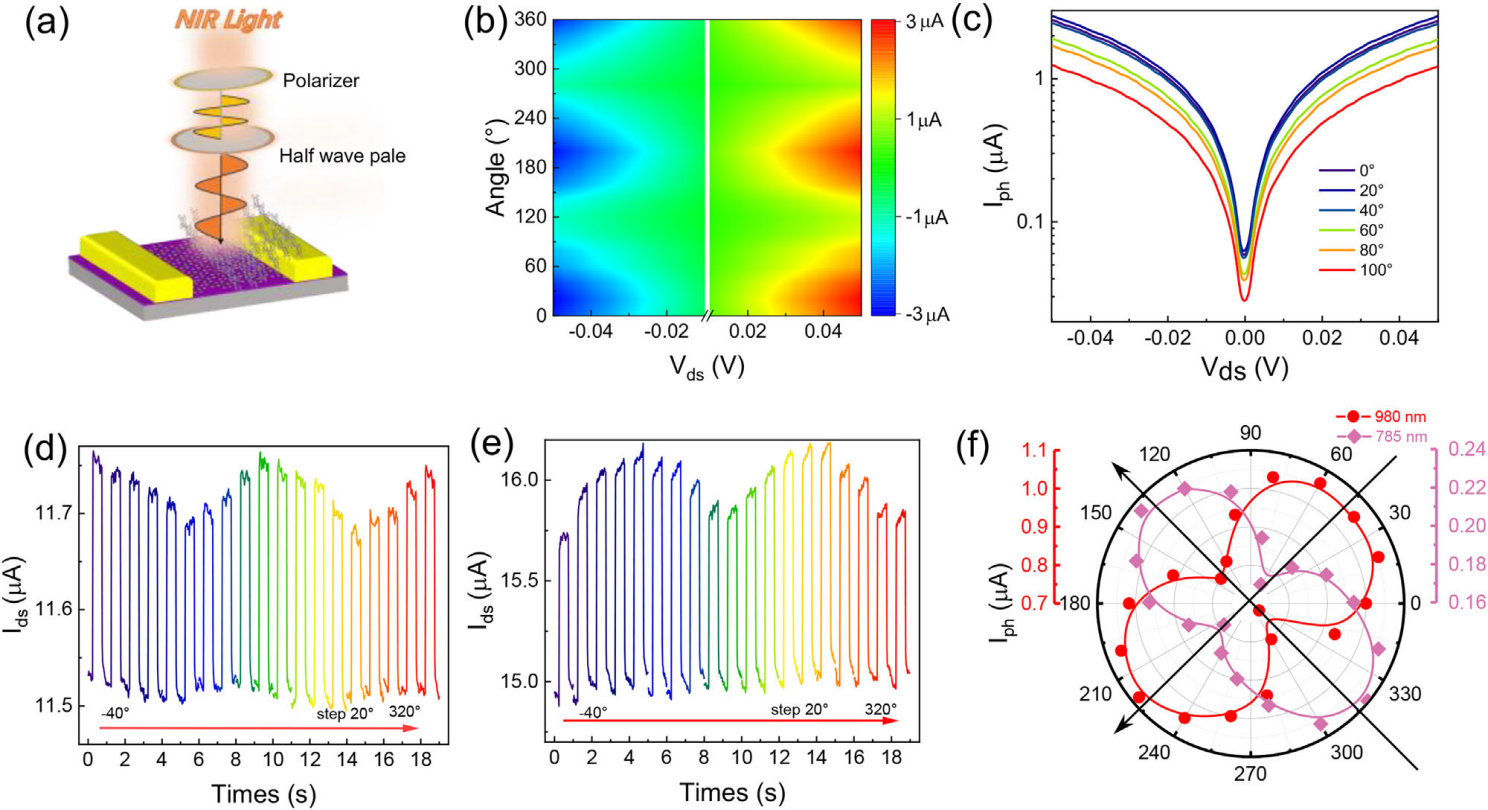
The linear polarization characteristics of the TiOPc/graphene heterojunction. a) Schematic diagram of a linear polarized light testing system. b) The polarization contour map of the photocurrent as a function of drain‐source voltage at various angles of TiOPc/graphene under 850 nm light illumination (V_g_ = 0 V). c) The polarization curves of the photocurrent generated by TiOPc/graphene as a function of the source‐drain voltage at a fixed angle under 850 nm light illumination d) Temporal response for 785 and e) 980 nm illumination under different polarization angles. f) Experimental polarization‐sensitive photocurrents are plotted with the linear‐polarization laser of 785 and 980 nm in the polar coordinates.

The TiOPc single crystal exhibits pronounced anisotropy, making it a highly promising candidate for designing polarization‐sensitive near‐infrared detectors. To investigate the photoelectric polarization characteristics of the TiOPc/graphene heterostructure device, a linear polarizer and a half‐wave plate were integrated into the test optical system, as shown in **Figure** **4**a. The incident light angle was adjusted by rotating the half‐wave plate. To confirm the polarization‐sensitive characteristics of the device in the near‐infrared band, a series of tests were conducted using near‐infrared lasers such as at 785, 850, and 980 nm. Figure 4b displays the 2D contour color map of the angle‐resolved photocurrent of the device under a bias voltage ranging from ‐50 to 50 mV during 850 nm light irradiation. The waveform surface plot indicates a strong dependence of the photocurrent on the polarization angle, with polarization sensitivity increasing with bias voltage. We further extracted the dependence curves of photocurrent on bias voltage from 0° to 180° polarization angles (Figure 4c), in which the photocurrent shows a clear polarization‐angle dependence. This phenomenon originates from the synergistic effect of the anisotropic structure of the TiOPc crystal. To further investigate the polarization performance of the device, a polarized temporal photoresponse test was performed to evaluate the photocurrent dichroic ratio (*DR* = *I*_max _/*I*_min _, where I_max_ denotes the maximum value of the photocurrent and I_min_ denotes the minimum value of the photocurrent), which is a critical parameter for assessing the polarization performance. By alternating the on/off states and synchronously rotating the half‐wave plate in 20° increments from 0° to 360° under a bias voltage of V_ds_ = 50 mV, the dynamic optical response of the device was continuously recorded. The dynamic optical response curves (Figure 4d,e) reveal that the photocurrents under 785 and 980 nm illumination exhibit phase‐opposite modulation characteristics: when the photocurrent for 785 nm reaches its peak, the response is at a minimum for 980 nm irradiation. To determine the photocurrent DR, we constructed a polar plot, as shown in Figure 4f. We can see that the photocurrents at the wavelengths of 785 and 980 nm exhibit a gourd‐shaped double‐lobe distribution with an ≈90° phase difference between their polarization directions. This phenomenon have been reported in other organic or inorganic material systems,^[^ ^31^, ^49^, ^50^ ^]^ which should be related to the polarization‐dependent absorption of TiOPc single crystal and dipole moment transition effects. Indeed, this phase difference is a gradual change with irradiation wavelength. We carried out the polarized photocurrent measurements under the 850 nm laser illumination (see Figure S13, Supporting Information), and the maximum response axis lies between the response‐line @785 nm and the response‐line @980 nm. From the anisotropic absorption of the TiOPc single crystal (Figure S13d, Supporting Information), we can see that the absorption intensity at 785 nm gradually increases with the polarization angle, while the absorption intensity at 980 nm exhibits an almost opposite trend. The dichroic ratios for 785 and 980 nm are calculated to be ≈ 1.40 and 1.48, respectively, demonstrating excellent anisotropic performance. The significant anisotropic performance obtained experimentally highlights the potential of this device for application in polarization‐sensitive near‐infrared detection, providing both theoretical foundations and experimental evidence for the development of novel anisotropic optoelectronic devices.

**Figure 5:**
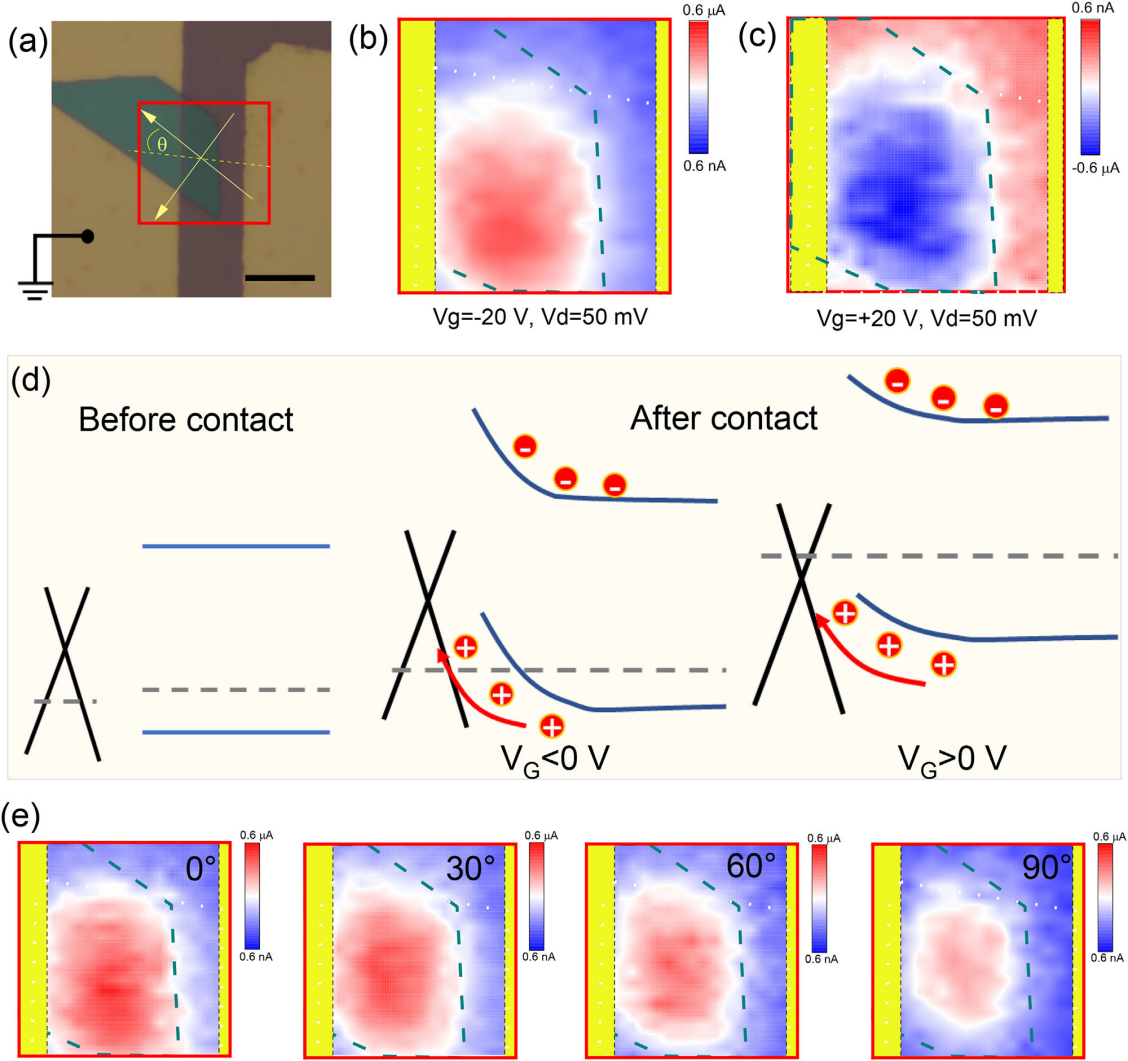
Photoelectrical characteristics of the asymmetric device. a) Optical image of the under test TiOPc/graphene device. Scale bar: 10 µm. b,c) Scanning photocurrent images measured under 50 mV bias with different gate bias. d) Schematic band diagrams of the device at 50 mV bias and different gate biases. e) Scanning photocurrent images measured under different polarization angles (0°, 30°, 60° and 90°).

**Figure** **5**a presents the microscopic image of a typical asymmetric device formed by the coupling of TiOPc and graphene, where only a portion of the graphene is covered by the TiOPc single crystal. To investigate the photocurrent generation mechanism in this asymmetric device, an 850 nm laser was employed to irradiate the coupled and overlapped region of TiOPc and graphene to obtain photocurrent mapping. The scanning area is marked with a red rectangle, as depicted in Figure 5a. As previously established, by exploiting the gate‐tunable work function properties of graphene, the intensity of the built‐in electric field can be dynamically adjusted through combined electrical and optical stimulation. Subsequently, we performed an in‐depth analysis to explore the control of the built‐in electric field using the gate bias. Photocurrent signals were measured under various gate voltages while maintaining a fixed source‐drain bias (V_ds_ = 50 mV), as shown in Figure 5b,c. The scanned photocurrent image reveals that photocurrent generation is primarily confined to the overlap between TiOPc and graphene, rather than across the entire channel, confirming the critical role of the TiOPc single crystal. This result can be attributed to the following: Photonic excitation induces the generation of a substantial number of electron‐hole pairs within the TiOPc crystal, which subsequently diffuse to the TiOPc/graphene heterojunction interface and separate into free carriers. The photogenerated holes transfer to the graphene channel due to the influence of the built‐in field and contribute to the photocurrent under the external electric field. For a negative gate voltage condition, the concentration of hole carriers would be enhanced, forming a consequently positive photocurrent in the TiOPc/graphene overlapping region in Figure 5b. On the contrary, the concentration of electron carriers dominates the conducting channel, leading to a negative photocurrent in Figure 5c. By applying different gate voltages, external electric field modulation induces a monotonic band bending in the conduction channel, thereby enabling deterministic control over the polarity of the photocurrent. Considering the intrinsic p‐type characteristics of graphene, a negative gate voltage bias increases the density of majority carriers, thus enhancing the band bending, as illustrated in Figure 5d. Therefore, a higher photocurrent is always observed under the negative gate voltage (Figures 2e and 3c). Comparable photocurrent results have also been achieved in other asymmetric devices, as presented in Figure S14 (Supporting Information), which underscores the universality of this design configuration. Figure 5e presents the photocurrent images at various polarization angles (the definition of the deflection angle is illustrated in the marked region, Figure 5a). It is evident that the photocurrent intensities exhibit a progressively decreasing trend from 0° to 90°, thereby providing further confirmation of the high polarization sensitivity of the TiOPc/graphene heterojunction.

**Figure 6:**
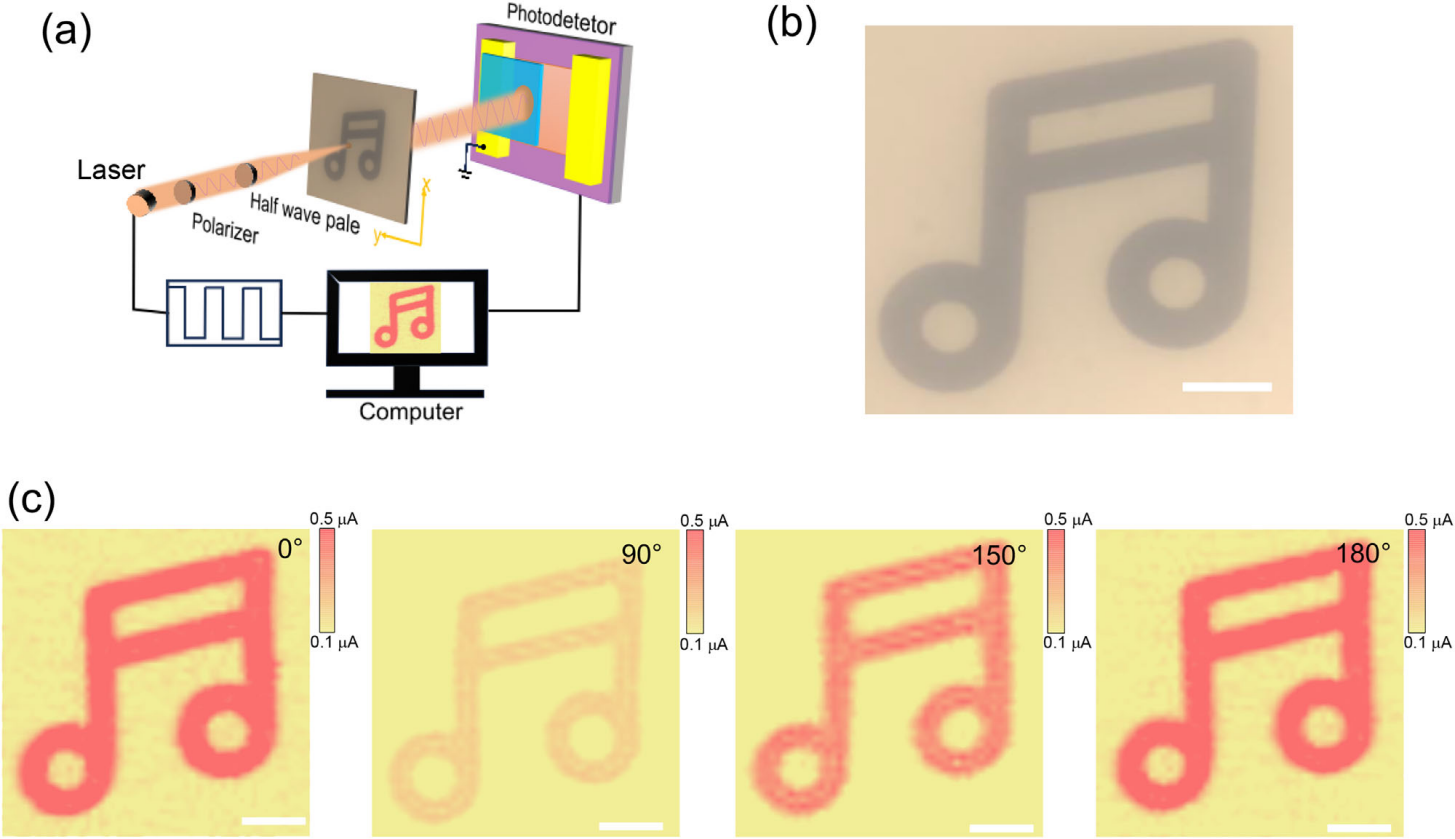
The application of polarization imaging. a) Schematic diagram of the polarization single‐pixel imaging measurement system. b) Optical microscope image of the object under test. c) The photocurrent imaging results of the note‐shaped object obtained under different polarization angles (0°, 90°, 150° and 180°). Scale bar: 20 µm.

High‐speed imaging functionality represents one of the most critical applications of advanced photoelectric detectors, and the significance of photoelectric detection and imaging capabilities has garnered substantial attention. Among these capabilities, polarization detection imaging plays a pivotal role across various domains, including military reconnaissance for identifying camouflaged targets and enhancing detection in complex environments, biomedical applications such as disease diagnosis and surgical navigation, and remote sensing tasks like geological exploration and ocean monitoring.^[^ ^51^, ^52^, ^53^ ^]^ To investigate the image sensing performance of this device in the near‐infrared band, we evaluated its polarization imaging capability using a single‐pixel scanning imaging system equipped with an independently constructed adjustable linear polarization angle. **Figure** **6**a presents the schematic diagram of the experimental setup for the polarization imaging equipment, where a 200 Hz laser signal serves as the frequency modulation source and is integrated into our polarization system. As a demonstration image, a metal mask is firmly mounted at the center of the 2D control platform, defining a high‐resolution scanning area of 100 × 100 µm^2^, with an initial step size of 2 µm. The diameter of the laser spot in the photocurrent imaging experiment is about 1.5 µm, which is sufficient for accurate target recognition. By controlling the movement of the 2D control platform, variations in photocurrent were induced, and the corresponding photocurrent values were systematically recorded. These current values were subsequently transformed into a current contrast mapping image. The optical image of the pattern on the metal mask is presented in Figure 6b. Figure 6c demonstrates the single‐pixel imaging results under several different polarization angles (0°, 90°, 150°, and 180°) using 850 nm near‐infrared light under a V_ds_ of 50 mV, employing an identical color scale for all imaging. From the color contrast, it can be observed that when the polarization direction is parallel to the long axis of the crystal, the image is the clearest; conversely, the image becomes less distinct when the polarization direction is perpendicular to the long axis of the crystal. The acquired images exhibit high spatial fidelity to the original mask pattern geometry, thereby confirming their robust imaging differentiation capabilities. These findings indicate that the organic single‐crystal heterostructure device is capable of performing high‐resolution video frame rate imaging.

## 3 Conclusion

In summary, we present an asymmetric organic heterostructure device featuring graphene as the carrier transport layer and TiOPc crystal as the light absorption layer. The device exhibited remarkable visible‐NIR photoresponse, including an excellent NIR responsivity of > 10^3^ A W^−1^ at 980 nm, a specific detectivity of > 10^10^ Jones and a ‐3 dB bandwith of > 1 kHz. Even under 1550 nm laser illumination, the responsivity reaches 32 A W^−1^, with a fast response speed (rise time of 250 µs), demonstrating the competitive overall performance as compared to previous organic polarization photodetectors. The zero bandgap of graphene leads to high dark current, restricting the specific detectivity to the level of 10^10^ Jones, which represents a performance gap compared to state‐of‐the‐art commercialize detectors. The excellent polarization characteristics were examined by Raman spectra and PL intensity, enabling strong NIR polarized response, including a photocurrent dichroic ratio of 1.48 for 980 nm irradiation and polarization‐dependent imaging. The physical mechanism of photocurrent generation was further elucidated through using photocurrent mapping. The device is capable of achieving high‐resolution video frame rate imaging in the near‐infrared band and holds potential for applications in polarized information security and high‐speed polarization imaging.

## 4 Experimental Section

4.1

4.1.1

##### 4.1.1.1 Preparation of Materials and Fabrication of Graphene/TiOPc Heterojunction Organic Transistors

TiOPc was purchased from MACKLIN and used without further purification. The TiOPc single crystal was synthesized via the micro‐distance air sublimation method at a growth temperature of 385 °C for 35 min. Initially, graphene was deposited on a Si/SiO_2_ substrate using the mechanical exfoliation technique with adhesive tape. Subsequently, two gold (Au) electrodes were precisely aligned and transferred onto the graphene layer using a microprobe under an optical microscope, resulting in the formation of a graphene field‐effect transistor (FET). Finally, the TiOPc crystal was partially overlaid onto the graphene channel through a dry transfer process utilizing polydimethylsiloxane (PDMS), thereby constructing an asymmetric organic photodetector based on the graphene/TiOPc heterojunction.

##### 4.1.1.2 Comprehensive Characterization of Material Microstructure

At room temperature and ambient conditions, atomic force microscopy (AFM) was performed using the Dimension Icon. Optical characterization protocols were developed by integrating cross‐polarized microscopy (POM) imaging using a Zeiss Imager A2m platform. These protocols incorporated wavelength‐resolved spectroscopic analyses and utilized a 532 nm laser excitation source for simultaneous Raman scattering detection and photoluminescence (PL) spectral acquisition.

##### 4.1.1.3 Electrical and Photoresponse Measurements

Time‐domain modulated photocurrent spatial profiling was performed under 850 nm laser excitation, using a square‐wave signal generator to investigate the dynamic carrier transport characteristics. This was achieved by precisely controlling the movement of a custom‐built 2D planar mobile platform while simultaneously capturing the photocurrent using a Keithley 6482 dual‐channel nanocoulombmeter/picoammeter. Electrical measurements were conducted at room temperature and under ambient conditions, utilizing both the Keithley 6482 and a Keithley 2400 SourceMeter for comprehensive parameter analysis. Optical responses were characterized using laser diodes with wavelengths of 785, 850, and 980 nm. The noise current was measured using the FS‐Pro semiconductor parameter analyzer (Primarius Technologies Co., Ltd., China).

## Conflict of Interest

The authors declare no conflict of interest.

## Supporting information



Supporting Information

## Data Availability

The data that support the findings of this study are available from the corresponding author upon reasonable request.
