# Supplementary material for: Highly‐Polarized Near‐Infrared Photodetector Based on 2D Organic/Inorganic Van Der Waals Heterostructure
Source: Adv Sci (Weinh). 2025 Aug 13;12(42):e08332. doi: 10.1002/advs.202508332 (PMC12622459; doi:10.1002/advs.202508332)
Supplement: Supplementary file 1 — Supporting Information [file ADVS-12-e08332-s001.docx]

**Supporting Information**

**Highly-Polarized Near-Infrared Photodetector Based on 2D Organic/Inorganic Van Der Waals Heterostructure**

*Wen Xu, Shuchao Qin***, Qianqian Du*, Yongji Zhang, Yanxun Zhang, Wenjun Wang, and Fengqiu Wang**

W. Xu, Dr. S. Qin, Dr. Q. Du, Prof. W. Wang

School of Physical Science and Information Engineering, Liaocheng University, Liaocheng 252059, China

E-mail: lcqinshuchao@126.com; dzdq0126@163.com

Prof. F. Wang

National Laboratory of Solid State Microstructures and Jiangsu Provincial Key Laboratory of Advanced Photonic and Electronic Materials, School of Electronic Science and Engineering, Nanjing University, Nanjing 210093, China

E-mail: fwang@nju.edu.cn

Keywords: TiOPc Single Crystal, Graphene, Heterostructure, Near-Infrared Photodetector

**Contents**

S1. TiOPc single crystal

S2. The PL of the device at different polarization angles

S3. Optical characterization of the uniformity of TiOPc single crystals and TiOPc/graphene

S4. The photoluminescence spectrum of TiOPc

S5. TiOPc/graphene hybrid device

S6. AFM images of TiOPc/graphene

S7. The optoelectronic performances for 850 nm

S8. The optoelectronic performance under 532, 658, 785 nm lasers

S9. The near-infrared response of another device

S10. The short-term stability of the device

S11. The response speed of device for 850 nm and 980 nm illumination

S12. Performance comparison of the device with other inorganic-based systems

S13. The alteration in the polarization orientation of the device

S14. Photocurrent mapping of another device

**S1. TiOPc single crystal**

**Figure S1.** Cross-polarized optical microscopy images of a TiOPc single crystal in different polarization directions. Scale bar: 20 μm.

**S2.** **The PL of the device at different polarization angles**

**Figure S2.** (a) The polar coordinate diagram of the 926 nm peak at different polarization angles. (b) The polar coordinate diagram of the 955 nm peak at different polarization angles.

**S3. Optical characterization of the uniformity of TiOPc single crystals and TiOPc/graphene.**

**Figure S3.** (a) Optical microscopy image of a TiOPc single crystal. (b-c) Photoluminescence (PL) and Raman mapping of the TiOPc single crystal. (d) Optical microscopy image of the TiOPc/Graphene heterojunction. (e-f) Photoluminescence (PL) and Raman mapping of the TiOPc/Graphene heterojunction. Scale bar: 20 μm.

**S4. The photoluminescence spectrum of TiOPc**

**Figure S4**. (a) Optical microscopy image of a TiOPc single crystal before transfer, and this TiOPc/Graphene heterojunction after transfer processing. (b) Comparative photoluminescence (PL) spectra of the TiOPc single crystal before and after transfer. Scale bar: 20 μm.

**S5. TiOPc/graphene hybrid device**


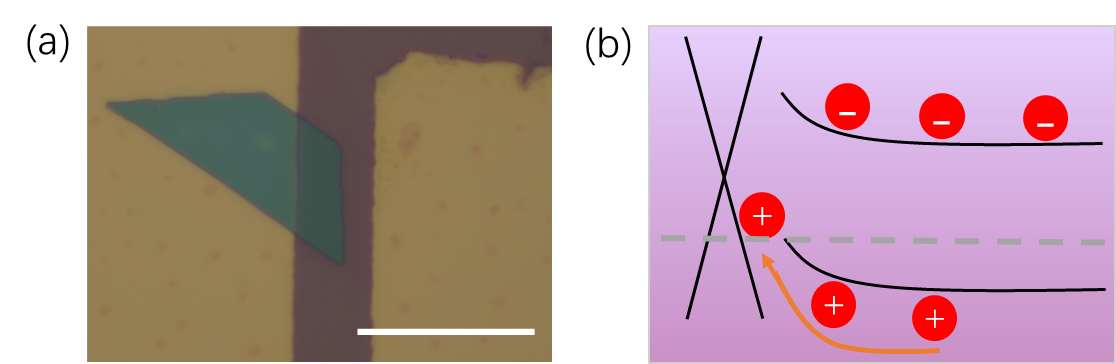


**Figure S5.** (a) Optical microscopy image of the TiOPc/graphene device. Scale bar: 20 μm. (b) Schematic illustration of charge transfer at the TiOPc/graphene interface.

**S6. AFM images of TiOPc/graphene**

**Figure S6**. (a) AFM image of the TiOPc/graphene interface. (b) Height profile image of the heterostructure interface. (c) KPFM image in dark. (d) Potential profile under dark. (e) The corresponding KPFM image under light illumination (λ = 980 nm). (f) Potential profile under light illumination.

**S7. The optoelectronic performances for 850 nm**

**Figure S7.** (a) Transfer curves of the TiOPc/graphene transistor under different optical powers (λ = 850 nm, V_d_ = 0.05V). (b) Responsivities as a function of light powers under different gate voltages (V_d_=50 mV). (c) The specific detectivity (D*) as a function of gate voltage (V_g_) at finite illumination (P=0.13 nW, V_ds_=0.05 V).

**S8. The optoelectronic performance under 532, 658, 785 nm lasers**

**Figure S8.** (a-c) Responsivities as a function of light powers under different gate voltages (λ = 532, 658 and 785 nm, V_d_=0.05 V).

**S9. The near-infrared response of another device**

**Figure S9.** (a-c) Responsivities as a function of light powers under different gate voltages (λ = 785, 850 and 980 nm, V_d_=0.05 V)

**S10. The short-term stability of the device**

**Figure S10.** Time-resolved photocurrent response measured under continuous 980 nm illumination for 15 hours at a modulation frequency of 1 Hz (λ = 980 nm, Vd = 50 mV).

**S11. The response speed of device for 850 nm and 980 nm illumination**

**Figure S11.** (a-b) The rise and decay times of the normalized photocurrents (λ = 850 and 980 nm).

**S12. Performance comparison of the device with other inorganic-based systems.**

**Figure S12.** Comparison of the responsivity of the device with other inorganic-based systems.

**S13. The alteration in the polarization orientation of the device**

**Figure S13.** (a-c) Experimental polarization-sensitive photocurrents are plotted with the linear-polarization laser of 785, 850 and 980 nm in the polar coordinates. (d) Polarization absorption characteristics in the wavelength range of 750 nm to 1000 nm under different polarization angles.

**S14.** **Photocurrent mapping of another device**

**Figure S14.** (a) Optical image of the TiOPc/graphene device. (b) Scanning photocurrent images measured at zero bias. (c) Scanning photocurrent images measured under 50 mV bias with different gate voltages. Scale bar: 10 μm.
